# Data on acute malnutrition and mortality among under-5 children of pastoralists in a humanitarian setting: a cross-sectional Standardized Monitoring and Assessment of Relief and Transitions Study

**DOI:** 10.1186/s13104-019-4475-x

**Published:** 2019-07-19

**Authors:** Emmanuel Nene Odjidja, Sonia Hakizimana

**Affiliations:** 1 Village Health Works, Kigutu, Burundi; 2 Present Address: AVSI Foundation, Torit, Eastern Equatoria South Sudan

**Keywords:** Malnutrition, Humanitarian mortality, Infant feeding practices, South Sudan, Pastoralists

## Abstract

**Objective:**

In humanitarian settings, children of pastoralists usually are the increased risk of malnutrition and its related complications. Consequently, as part of the program’s targeted response to the burgeoning malnutrition caseloads, a nutrition and mortality survey was conducted using a global standardized methodology in humanitarian settings in Ikwotos country of the Eastern Equatoria of South Sudan. Additionally, in understanding the intricacies of food diversity consumed in the households, we used infants as a proxy of household feeding and collected information on the range of foods consumed by households.

**Data description:**

Data contained in this note is a standard cross-sectional survey conducted in South Sudan with children between the ages of 6 and 59 months, although the mortality component covered all members of the household. While data for mortality and infant feeding practices were self-reported, the assessment of nutritional status were in accordance to the World Health Organisation’s guidelines for nutrition assessment. Age, sex, height and mid-upper arm circumference data were assessment and malnourished children were classified as those with Z-score between − 2 and − 3 and those above − 3 were classified as severely malnourished.

## Objective

Children of pastoralists demonstrate the highest forms of acute malnutrition globally compared to other agrarian societies in same countries or geographic areas [1–3]. A study conducted to assess the wasting levels as determined by weight for height status among children in the Greater Horn of Africa (Eritea, Kenya, Somalia, South Sudan and Uganda) showed a prevalence of 17%, counting to a 7% increase in children of pastoralists [4]. Nutritional status of pastoralists children have been dependent on several factors which spans from geographical position and its environment ramifications to cultural conception about food as well as general access to health services. Geographically, most pastoralists live in the arid and semi-arid regions of most countries which are most bedevilled with seasonal and intermittent droughts [5]. This situation triggers food insecurity in those regions which eventually culminates to predominantly high malnutrition rates. Regarding culture, pregnant pastoralists believe that restricting intake of some major micronutrients such as iron, folic acid and niacin eases delivery. This often increases the risk of small birthweight which is highly associated to acute and chronic malnutrition, child morbidity and mortality [6]. Exclusive breastfeeding is limited among pastoralists as milk is replaced with goat or cow milk within few days after delivery which is deficient in major micronutrients and increases the risk to malnutrition among infants [6, 7].

This Standard Monitoring and Assessment of Relief and Transitions (SMART) Survey was conducted in the Ikwoto county of South Sudan between September and December 2017 by AVSI Foundation, South Sudan to assess mainly the prevalence of malnutrition and mortality among children. The study was conducted among children aged 6–59 months which this age bracket is selected because of global recommendation that children in these ages have an increased risk to malnutrition and mortality [8].

## Data description

This is a cross-sectional study which collects information on nutrition of children under-5 specifically between 6 and 59 months. We used a predefined and standard survey which has been predeveloped, pretested and globally accepted benchmark for malnutrition and mortality assessment in humanitarian settings [9]. The data was collected enumerators and measurers who were grouped into teams. Enumerators were tasked with the responsibility of collecting general information regarding mortality, infant and child feeding whilst measurers conducted child anthropometric measurements [10, 11]. These anthropometric measurements aimed at assessing nutritional status of children and collected information on age, sex, height, weight and mid-upper arm circumference. For the mortality assessment, a comprehensive household information was collected which comprised of members of the household including those that died [11].

The dataset contained in the Microsoft Excel version 2013 file in Table 1 has eight tabs. The first tab contains a complete tab of household demographics with the second containing details of individuals in each household. Tab three and four are disaggregation between members of the household who left 6 months to the survey and those who died respectively. Tab five and six are the raw and cleaned version of the anthropometric data collected which contains the nutritional parameters as described. The final two tabs are details of raw and cleaned versions of details of infant and young child feeding practices.

**Table 1:** Overview of data files/data sets

Label	Name of data file/data set	File types (file extension)	Data repository and identifier (DOI or accession number)
Data file 1	Primary dataset of survey containing eight tabs described above	MS Excel file (.xlsx)	10.3886/E108684V3
Supplementary material 1	Complete report of nutrition survey conducted	Pdf file (.pdf)	10.3886/E108684V3
Supplementary material 2	Backup of primary dataset file	.bak file (Backup file)	10.3886/E108684V3

The first supplementary material contained in Table 1 is a preliminary report of the study which presents a descriptive overview of the state of nutrition and mortality in the study area. Additionally, the report presents contextual information of current nutritional and under-5 program activities carried out by health partners in the study area along with justification for this study.

The second supplementary material in Table 1 is a backup of the primary dataset which can be accessed using a Microsoft Structured Query Language (SQL) server.

### Outcome variables

Variables measured in this data note includes nutritional outcomes among children under 5, behavioural related variables on infant and child feeding along with general and under 5 mortality figures. These variables are further explained below:

In assessing malnutrition among children, two main outcomes were considered which is in accordance to the World Health Organisation’s standards [12]: acute and chronic malnutrition.

Acute malnutrition (wasting) was classified as weight-for-height status of children under five and chronic malnutrition (stunting), classified as height-for age.

### Sampling

The study employed a 2-stage sampling strategy comprising of cluster and simple random sampling. Clusters were selected using a probability proportional to population size (PPS) and for the second stage, we listed all households in the selected cluster and randomly selected households using generated random numbers. Sample size of 2222 persons and 433 households was calculated which was divided among a cluster size of 30. This was derived from an assumed parameter for global malnutrition prevalence of 16.9% of the catchment area, 95% confidence and 80% power to detect a 25% higher global acute malnutrition prevalence [13].

## Limitations

The dataset has some notable limitations which should be considered when reviewing or reanalysing. Given the fast changing landscape of humanitarian settings like South Sudan, this quantitative data might not necessarily give a complete overview of the complexities that underpins nutrition and mortality in the study area. Therefore to increase the suitability to inform nutrition programming, it is recommended that further qualitative investigation is conducted to further explore the emanating findings in this study. Furthermore, this exploratory study will bring more useful information about the context and how this creates various generative mechanisms, leading to the outcomes measured in this study.

Secondly, this is a cross-sectional data with no control group and therefore, cannot claim causality. This implies that statistical inferences and conclusions drawn from this data especially from statistical modelling should be cautious in making general “cause and effect” relationships.

In addition, the dataset on infant and young children feeding practices as well as mortality were self-reported and possibly, could have been either overestimated or underestimated in the study context. Triangulating these results with a field qualitative investigation will provide more useful contextual information on mortality and infant and young children feeding practices.

## Data Availability

The data described in this note can be freely and openly accessed on ICPSR repository: 10.3886/E108684V3 also see Table 1 details on data accessibility.

## Abbreviations

MUAC: mid-upper arm circumference
PPS: probability proportional to population size
UNICEF: United Nations Children’s Fund
WHO: World Health Organisation
SMART: Standardized Monitoring and Assessment of Relief and Transitions
SECA: SECA GmbH & Co. KG
SQL: Structured Query Language
